# The Clinical Utility of Whole Body Vibration: A Review of the Different Types and Dosing for Application in Metabolic Diseases

**DOI:** 10.3390/jcm13175249

**Published:** 2024-09-05

**Authors:** Abigayle B. Simon, Pratima Bajaj, Joe Samson, Ryan A. Harris

**Affiliations:** 1 Georgia Prevention Institute, Medical College of Georgia, Augusta University, Augusta, GA 30912, USA; absimon@augusta.edu (A.B.S.); pbajaj@augusta.edu (P.B.); 2 Department of Medical Illustration, Augusta University, Augusta, GA 30912, USA; jsamson@augusta.edu

**Keywords:** vibration, metabolism, diabetes, obesity

## Abstract

Whole body vibration (WBV) is an innovative exercise mimetic that utilizes a vibrating platform to transmit mechanical vibrations throughout the body. WBV has been a popular area of research in recent years due to its potential physiological and therapeutic benefits in both health and disease. The utility of WBV is rooted in the various parameters (i.e., frequency, amplitude, duration) that affect the overall dose of vibration delivered to the body. Each type of WBV, coupled with these aforementioned parameters, should be considered when evaluating the use of WBV in the clinical setting. Thus, the purpose of this review is to provide an overview of recent literature detailing the different types of WBV, the various parameters that contribute to WBV efficacy, and the evidence of WBV in metabolic disease. A systematic search was conducted using Medline, Embase, Cochrane, CINAHL, and PubMed. All types of study designs were considered, with exclusions made for animal studies, duplicates, and study protocols without data. Thirty-four studies were included. In conclusion, as a modern exercise mimetic with therapeutic potential for metabolic diseases, understanding the interplay between the types and dosing of WBV is critical for determining its utility and efficacy. Further studies are certainly needed to elucidate the full therapeutic potential of WBV in metabolic diseases.

## 1. Introduction

Whole Body Vibration (WBV) has emerged as an exercise mimetic in recent years and represents an innovative and easily accessible exercise modality that has gained significant popularity in both research and personal use. WBV involves utilizing a vibrating platform to expose the body to mechanical vibrations, leading to various physiological responses that have contributed to the documented benefits following use. During vibration, skeletal muscles rapidly contract and relax at a certain frequency, which promotes muscle anabolism and improved stretch reflex through the activation of muscle spindles [1]. Additionally, WBV promotes an increase in nitric oxide production [2] and enhances the circulation of systemic blood flow, which contributes to increased oxygen delivery to organs and tissues throughout the body. WBV can also activate the endocrine system and increase the production of testosterone and various growth hormones, each crucial for maintaining skeletal muscle mass [3]. Furthermore, WBV promotes increases in bone density, stimulates bone remodeling [4,5], and has the potential to enhance neuromuscular function, leading to improvements in balance and coordination [6]. In fact, a reduction in pain has been reported following WBV, which may be attributed to its inhibitory effect on neurons of the spinothalamic tract [7]. Collectively, WBV represents an innovative exercise alternative that has therapeutic potential in metabolic disease and in circumstances in which individuals are unable to perform traditional types of exercise (i.e., walking, running, cycling, etc.). There are, however, different parameters of WBV that should be considered, including both frequency and amplitude of the elicited vibration. Each of these parameters affects the intensity and dose of the delivered WBV, and different doses can provide distinct advantages in various patient populations. This review will provide an overview of the different types of WBV and the dose parameters that contribute to the efficacy of use in metabolic disease.

## 2. Methods

A comprehensive review of the literature to date was undertaken to explore the diverse modalities of whole-body vibration (WBV) and the specific dosage parameters available. The search encompassed databases including Medline, Embase, Cochrane Central Register of Controlled Trials, Cochrane Database of Systematic Reviews via Ovid, and CINAHL via Ebsco and PubMed. Relevant articles were identified using Medical Subject Headings (MeSH) such as “frequency”, “amplitude”, “diabetes”, “metabolic disease”, “osteoporosis”, and “obesity”, combined with the term “whole-body vibration”, with consistent adjustments made for various databases. All types of study designs were considered, with exclusions made for pre-clinical studies, duplicates, and published study protocols with no data. Manuscript abstracts deemed relevant and requiring further evaluation underwent full-text review, and eligible studies were included for evaluation. Thirty-four studies were included in the review, distributed among the various metabolic diseases as follows: ten studies for osteoporosis, ten studies for overweight and obesity, eight studies for type 2 diabetes, and six studies for metabolic syndrome. When provided by the referenced study, the type, frequency, and amplitude of the WBV plate that was used are given in the text below. Screening, publication review, and investigation summary were independently performed by two investigators (ABS and PB), following the PRISMA (Preferred Reporting Items for Systematic Reviews and Meta-Analyses) checklist. Discrepancies were resolved through consultation with the senior author (RAH).

## 3. Types of Whole Body Vibration

There are various types and styles of WBV that exist on the market today, each offering a unique form of vibration delivery that ranges from personal to commercial use. The following section will elaborate on these various types of vibration devices and report the advantages and disadvantages associated with each.

### 3.1. Vertical (Linear) Vibration

**Figure 1:**
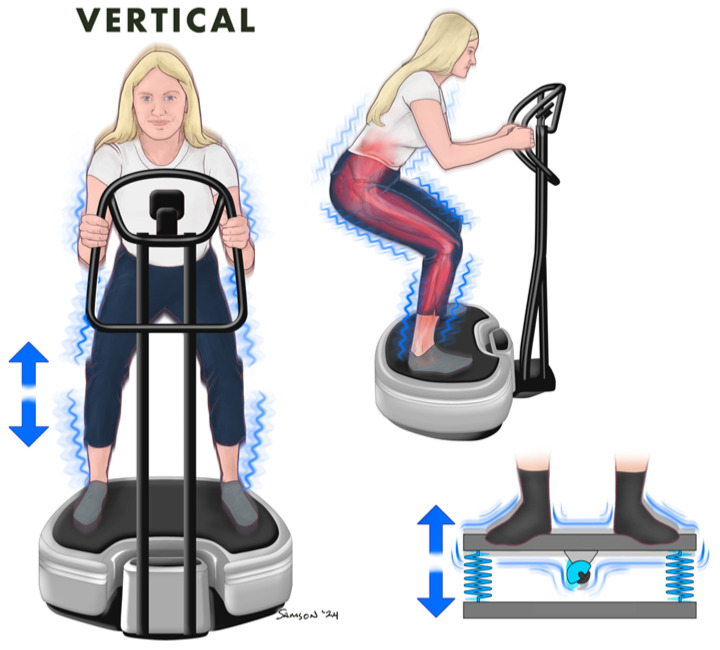
Vertical Whole Body Vibration.

Vertical WBV utilizes a vibration device that operates by generating up-and-down oscillations, typically ranging from 0.1–7.5 mm of travel, along a vertical axis. Participants position themselves on the platform, ensuring their feet are shoulder-width apart, knees flexed at approximately 130 degrees, and hands resting lightly on the handlebars (Figure 1). The vibration patterns induced are very stable as they move vertically with minimal horizontal vibrations. In addition, simultaneous and symmetrical movement of both sides of the body is initiated during vertical WBV exposure. In fact, vertical WBV has been favored and found to be more comfortable by individuals compared with other types of WBV, especially given that the platform aligns with gravity’s natural direction and does not expose the body to unnatural forces [8]. Accordingly, vertical WBV could be beneficial to individuals with musculoskeletal injuries; however, it may not be suitable for everyone, particularly for individuals who experience neck pain or vertigo [9]. Side effects that have been reported following vertical WBV include reduced capacity for visual-motor monitoring and reduced visual acuity [8]. Additionally, vertical WBV induces thoracic vibration, thus resulting in potential voice changes while on the platform (Figure 1). Nonetheless, vertical WBV platforms are widely used throughout research and personal use, yet there appear to be greater risks involved with respect to physical functioning that should be considered prior to use.

### 3.2. Rotational (Pivotal/Teeter-Totter/Oscillating) Vibration

**Figure 2:**
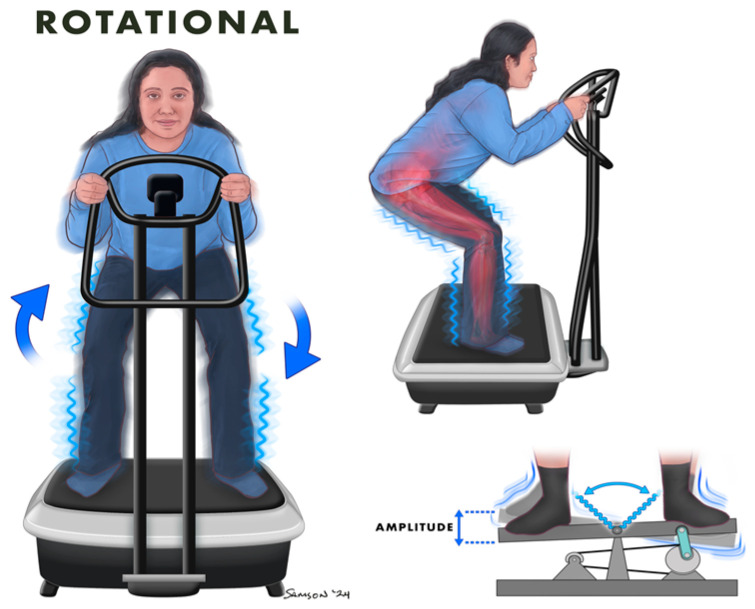
Rotational Whole Body Vibration.

Rotational WBV generates lateral accelerations and vertical vibrations by alternately displacing the left and right sides around a central pivot point (Figure 2), increasing both amplitude and lateral acceleration potential compared to vertical WBV. Similar to the stance utilized in vertical WBV, individuals ensure their feet are shoulder-width apart, knees flexed at approximately 130 degrees, and hands resting lightly on the handlebars (Figure 2). During rotational WBV, the vibration amplitude is dependent on the foot placement on the platform. Greater amplitude is achieved with the feet spread wider apart as this position increases the distance from the central axis of rotation [8,10]. Indeed, rotational WBV has demonstrated effectiveness in enhancing balance, making it a potentially more suitable platform for older individuals who are at an increased risk of falls [11]. Given the imprecise method of changing amplitude, rotational WBV is less frequently employed in research. However, the associated risks with rotational WBV may be lower, given the ability to minimize head acceleration and dampen the transfer of mechanical energy to the spine and head during use [9].

### 3.3. Comparison of Vertical and Rotational Vibration

Differences in amplitude and frequency, which are both key parameters of WBV dosage, alongside muscular responses, are apparent between vertical (Figure 1) and rotational WBV (Figure 2). Twelve weeks of rotational WBV was found to engage a broader range of muscle groups and proprioceptive responses compared to control and vertical WBV training [8,10]. Given the natural hip rotation during normal patterns of walking gait, rotational WBV (Figure 2) exhibits the lowest risk and is recommended as a therapeutic for patients with chronic pain [8]. In addition, greater neuromuscular activity has been observed with rotational WBV compared with vertical WBV, and the recruitment of more motor units and greater muscle activation can increase the isometric activity of the muscles [8,12,13]. Further, rotational WBV (Figure 2) elicits higher amplitudes and transmits greater acceleration to different parts of the body compared with vertical WBV (Figure 1) [8]. In fact, a 71–189% greater transmission of vibration to the upper head and body has been reported following vertical WBV in healthy men and women compared with rotational WBV [9]. These data suggest that rotational WBV may be more suitable for use in individuals who experience neck pain or vertigo than vertical vibration. Nonetheless, for use of all types of vibration platforms, it is recommended to maintain the knees slightly bent (between 125–130 degrees) while standing on the platform to minimize head movement (Figure 1 and Figure 2) [9]. In conclusion, rotational WBV offers the least number of adverse effects [8,9] and activation of more muscle groups [14] compared to vertical vibration; however, when and the reason to use the specific type of WBV and the exact benefits of each are incompletely understood and warrants further investigation. 

### 3.4. Sonic Wave Vibration

**Figure 3:**
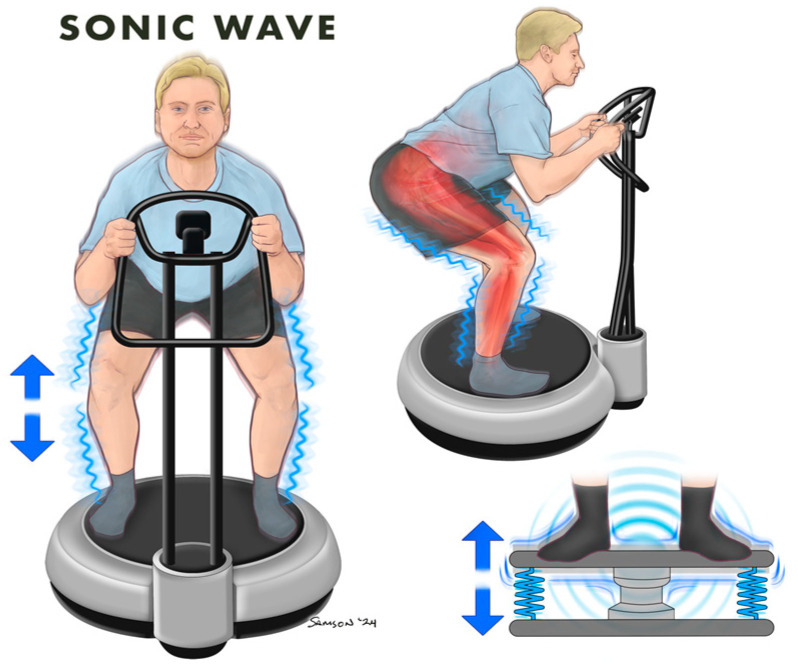
Sonic Wave Whole Body Vibration.

Sonic wave vibration (SWV) (Figure 3) is another form of WBV and refers to the use of sound waves to generate vibrations throughout the body. A recent study assessed the effect of SWV on the autonomic and cognitive function of elderly participants aged 88 ± 5 years. An increase in heart rate variability, an index of the parasympathetic nervous system, and resting energy expenditure were observed, while execution time during the Stroop test decreased following 8 weeks of SWV for 10 min/day, 5 days per week [15]. In addition, an increase in stability and pleasure levels of mood was noted following SWV. These findings indicate that SWV could be beneficial for elderly people as it may have positive effects on mood, the autonomic nervous system, cognitive function, and other cerebral benefits. Although SWV has not been used extensively, no adverse effects of SWV have been reported [15]. Little research has been conducted using SWV; therefore, findings from this provocative trial warrant further studies using this type of WBV. 

## 4. Dose of Whole Body Vibration

The dose of traditional modes of exercise can be adjusted by changing the time and intensity of the activity. Similarly, the dose of WBV can also be manipulated. Quantification of the dose of WBV requires three components: time, frequency, and amplitude. Time is the amount of time spent on the platform during active vibration. Frequency denotes the count of vibration cycles per second, essentially reflecting the speed of motion of the WBV platform, a parameter that is quantified in Hertz (Hz). Amplitude, on the other hand, characterizes the vertical displacement of the WBV platform throughout each vibration cycle, quantified in millimeters (mm). The amplitude serves as a gauge of WBV intensity. Specifically, a higher amplitude produces a more robust vibration, and a lower amplitude results in a milder vibration. The vibration dose (m/s^1.75^) for continuous, steady vibration can be calculated by the following equation: [(1.4a)^4^ × b]^14^, where a represents root mean square acceleration (m/s^2^) and b represents duration (s) [16]. The following sections will provide a comprehensive examination of the existing literature considering the variations in frequencies and amplitudes currently employed during WBV use.

### 4.1. Frequency of Vibration

The frequency of WBV is an important parameter that not only influences the therapeutic potential but also the physiological effects, necessitating careful consideration of the chosen frequency. In general, it is recommended that lower frequencies be used for rehabilitation purposes [17], whereas higher frequencies are used for neuromuscular activation [18]. Nonetheless, it is important to note that the majority of studies have utilized frequencies less than 50 Hz, while frequencies higher than 50 Hz may be associated with a greater risk of adverse events [19]. In fact, the most effective frequencies for improving skeletal muscle endurance and strength have been found to be around 30 Hz, with potential variations higher or lower depending on specific demographics and performance goals [19]. In the realm of muscle performance, six 60-s sets of WBV at a frequency of 30 Hz led to improved squat and countermovement jumps and quadricep power output [20]. Additionally, neuromuscular stimulation during squat jump and countermovement jump was notably impacted by frequency, where higher frequencies (50 Hz) were more effective in increasing average peak power compared to lower frequencies (20–35 Hz). However, there are conflicting data in healthy adults that found no changes in isometric muscular endurance or fatigue index following a single session of WBV (5 bouts of 60 s at 30 Hz) [21]. Perhaps intuitive, age has been a critical factor when determining which frequency of WBV to use, specifically among older adults. Compared with younger adults, older adults experienced greater muscle contraction following a WBV frequency of 20 Hz compared to 40 Hz [22]. Indeed, the optimal vibration frequency for older adult women was found to be 33 Hz, resulting in a 25.9% increase in maximal power output compared to frequencies of 20 Hz and 50 Hz [23]. In contrast, WBV using both 20 Hz and 40 Hz for 24 weeks resulted in similar changes in bone mineral density in elderly women matched for age, weight, and height [24]. The effect of modulating WBV frequency has also been shown in post-stroke rehabilitation. Higher frequencies resulted in greater concentric and eccentric muscle strength as well as reduced bone resorption [25]. These findings highlight the importance of choosing the appropriate WBV frequency to augment neuromuscular responses [26]. Indeed, the frequency of WBV can also affect vascular adaptations. A significant reduction in brachial-ankle pulse wave velocity was observed in healthy individuals following 30 Hz of WBV during a static squat position compared with lower frequencies (12–20 Hz) [27]. Collectively, these findings emphasize the role of frequency selection in tailoring WBV interventions to specific objectives and patient populations. In addition, these studies highlight the need for careful consideration of frequency selection based on the application and goals of therapy. Nonetheless, future studies are certainly needed to identify the most effective WBV frequency for each and every application.

### 4.2. Amplitude of Vibration

The amplitude of WBV is also an important parameter in determining the therapeutic effects and can be altered synergistically with frequency. Amplitude is defined as half the difference between the highest and lowest points of the oscillating platform. Amplitude plays an important role in skeletal muscle activation and neuromuscular adaptations. In a 6-week study conducted in physically active young adults, neuromuscular activation and lower limb hypertrophy were improved more following 4 mm displacement compared with 2 mm [28]. Indeed, higher frequency (50 Hz) and lower amplitude (0.5 mm) have been demonstrated to induce the largest neuromuscular activity during graded isometric contractions, surpassing combinations of higher amplitude (1.5 mm) with frequencies of either 30 Hz or 50 Hz [29]. In addition, a single bout of linear, low amplitude (2.5 mm) WBV (10 min; 30 Hz) in healthy men and women resulted in higher insulin sensitivity and muscle oxygen consumption and a lower inflammatory (IL-6) response compared to high amplitude (5 mm) [30]. Taken together, these findings suggest that both frequency and amplitude play a role in determining the most effective approach for induction of neuromuscular activity [29]. Moreover, body position during WBV appears to affect the electromyographic (EMG) response in leg muscles when testing different positions and vibration amplitudes. In middle-aged and older women, dynamic, semi-squat WBV training at an amplitude of 4 mm is recommended for enhancement in lower limb muscle strength and function compared to amplitudes of 0 mm and 2 mm [31]. Collectively, although some studies support the efficacy of higher amplitudes, the majority of studies demonstrate that lower amplitudes (≤2 mm) offer the greatest effectiveness. Nonetheless, future studies are certainly needed to identify the most effective WBV amplitudes for each and every application.

## 5. Whole Body Vibration in Metabolic Disease

The use of WBV in metabolic diseases is a relatively new area of research and may have substantial implications for improved clinical outcomes in the future. The following sections expand on the utility of WBV in patients with osteoporosis, overweight and obesity, type 2 diabetes, and metabolic syndrome.

### 5.1. Use of Whole Body Vibration in Osteoporosis

Research into the effects of WBV has illuminated its potential benefits across diverse patient populations, including those with osteoporosis. Encouraging outcomes emerged following a six-month vertical WBV (Figure 1) (30 Hz, 5 mm) study that was conducted in women diagnosed with osteoporosis. Compared with women without osteoporosis, treatment with WBV (5 sessions per week; 6 months; 30 Hz; 5 mm) resulted in a 4.3% increase in bone mineral density and a concurrent reduction in chronic back pain [32]. In men with osteoporosis, rotational WBV (6 months; 25.5 Hz) improved trunk extension strength and chair rise test scores compared to baseline [33]. Additionally, eight weeks of rotational WBV in older men (5 sessions per week; 12–16 Hz; 3–5 mm) showed significant improvements in muscle strength and timed-up-and-go test compared to control groups [34]. Further, in men with COPD coupled with osteoporosis, WBV (36 weeks; three sessions per week) improved both lung function, bone strength, and exercise capacity [35]. Similarly, an increase in spine and tibia bone mineral density was observed following vertical WBV (5 sessions per week; 6 months; 90 Hz; 0.1mm) in both children and young individuals, extending its therapeutic potential to a younger demographic [36]. Beyond bone health, the impact of WBV on balance and quality of life has also been documented. Eight months of vertical WBV (3 sessions per week; 12.6 Hz, 3 mm) was compared with walking (3 sessions per week; 55 min). The WBV group experienced a 29% increase in balance and a 4.3% increase in bone mineral density at the femoral neck, accentuating the potential of WBV to promote postural stability [37]. In addition, both vertical and rotational WBV (3 sessions per week for one year; 35 Hz, 1.7 mm) were demonstrated to alleviate larger joint pain, a significant determinant of quality of life for individuals with osteoporosis [38]. Indeed, WBV using frequencies >20 Hz coupled with extended WBV exercise duration may yield the most favorable results in people with osteoporosis, emphasizing the importance of a WBV protocol tailored to the individual’s needs [39]. Importantly, a WBV regimen of high frequency coupled with low amplitude has been well-tolerated among individuals with osteoporosis, and no adverse events have been documented [40,41]. Taken together, these findings highlight the multifaceted potential of WBV for individuals with osteoporosis, spanning from bone health to balance enhancement and alleviation of pain.

### 5.2. Use of Whole Body Vibration in Overweight and Obesity

WBV has also been used as an exercise mimetic in individuals with obesity (BMI > 30 kg/m^2^). Within an intervention lasting six weeks or more, substantial benefits have emerged in middle-aged, obese women, demonstrating improved cardiac autonomic function and a notable reduction in central and peripheral arterial stiffness. Notably, a 10-week vertical WBV intervention (2 sessions per week; 40–60 Hz, 2.0–5.0 mm) resulted in significant reductions in both weight and fat mass in obese women [42,43]. Additionally, a remarkable reduction in femoral-ankle pulse wave velocity was observed in obese individuals following eight weeks of vertical WBV (4 sessions per week; 25–40 Hz, 1–2 mm) [44]. Moreover, in both young and postmenopausal, overweight and obese women, vertical WBV (6 weeks; three sessions per week; 25–30 Hz, 1–2 mm) effectively lowered brachial systolic blood pressure, aortic blood pressure, and reduced arterial stiffness [45,46]. Eight weeks of WBV can also reduce body fat percentage and improve insulin sensitivity in middle-aged obese adults when compared to a control group without WBV [47]. In addition, greater body fat reduction was observed in sedentary obese participants following a combination of hypocaloric diet and WBV compared with diet alone, albeit both groups exhibited weight loss. Furthermore, those who incorporated WBV with diet not only exhibited a greater increase in the insulin sensitivity index—measured through oral-glucose-tolerance tests—but also reduced post-load glucose compared with the control group [47]. Moreover, a six-week WBV training protocol conducted three times weekly in obese, postmenopausal Hispanic women has demonstrated the capacity to significantly enhance heart rate variability, decrease body fat percentage, and increase muscle strength (both *p* < 0.01), emphasizing its potential to promote cardiovascular health [48]. Additionally, in overweight and obese men and women, a single bout of rotational WBV (10 min; 14 Hz, 2.5 mm) reduced circulating concentrations of endothelin-1, a potent vasoconstrictor that plays a critical role in the regulation of vascular tone [49]. Further, an acute bout of rotational WBV (10 min; 14 Hz, 2.5 mm) stimulated favorable metabolic and immune responses in individuals with obesity, as evidenced by improvements in circulating glucose and insulin, HOMA-β cell function, and lymphocyte normalization [50]. In addition, an acute bout of WBV (30 Hz) decreased circulating concentrations of triglycerides, providing data that support the efficacy of WBV in facilitating fat oxidation and inducing lipid mobilization in individuals with obesity [51]. In summary, the extensive body of research on WBV in overweight/obese individuals presents a compelling narrative of its multifaceted benefits. There is evidence to support an improvement in cardiac autonomic function, reduction in arterial stiffness, weight loss, reduction in body fat percentage, and enhancement in cardiovascular parameters in overweight and obesity, all following WBV. Accordingly, WBV stands as a promising exercise alternative for individuals with increased adiposity, offering a multifaceted approach to health and wellness, particularly in those who cannot engage in traditional forms of exercise.

### 5.3. Use of Whole Body Vibration in Type 2 Diabetes

Controversy pervades the literature concerning the potential impact of WBV on various outcomes in individuals with type 2 diabetes, including ambiguity in glycemic control, arterial stiffness, and body composition. In a 12-week trial utilizing a rotational WBV (Figure 2) (12–16 Hz; 4 mm) in elderly, obese individuals with type 2 diabetes, a reduction in hemoglobin A_1c_ (HbA_1c_) of 0.55% was observed [52]. Additionally, a similar rotational WBV protocol in individuals with type 2 diabetes resulted in reductions in body fat and waist circumference, as well as a concurrent increase in overall leg blood flow [53]. These findings have been sustained in longer trials of WBV as well. Notably, a 6-month study exploring the use of vertical WBV in older adults with type 2 diabetes documented significant improvements in hemoglobin A_1c_ and geriatric depression scores, with no hypoglycemic events reported during the study [54]. In support, maintenance of HbA_1c_ was observed following 12 weeks of vertical WBV (Figure 1) (30–40 Hz; 2–4 mm) in both men and women with type 2 diabetes, while an increase in HbA_1c_ was observed following a control condition without WBV [55]. These beneficial effects on glycemic status have been observed in acute settings as well. In a study that employed elderly women, both with and without type 2 diabetes, a single bout of WBV (1 session; 35 Hz; 4 mm) was effective at reducing circulating concentrations of glucose to levels comparable to those observed in healthy elderly women [56]. In addition, an 8-week intervention employing rotational WBV with an intermediate frequency and high amplitude (18.5 Hz and 4 mm, respectively) demonstrated significant decreases in body fat and improvements in lipid profile exclusively within individuals with type 2 diabetes compared to no differences observed in the control group [57]. Moreover, vertical WBV (3 sessions per week for 6 weeks; 12 Hz, 5 mm) may also have positive impacts in individuals with type 2 diabetes on overall quality of life with respect to pain perception, fatigue, and physical functioning [58]. Further, 12 weeks of rotational WBV (12–16 Hz; 4 mm) was not only well-tolerated, but it also enhanced balance among elderly individuals with type 2 diabetes and could potentially reduce the risk of falls [11]. Conversely, an 8-week intervention with WBV (3 sessions per week; 30 Hz, 2 mm) resulted in no changes in BMI or body fat [59]. Thus, the use of WBV in individuals with type 2 diabetes has shown promise in improving clinical outcomes, including HbA_1c_, glycemic control, blood flow, and body composition. It is important to note that the type of WBV and frequency and amplitude of the aforementioned studies were not standardized and could certainly impact the outcomes. Further, while lower frequencies (between 12–18.5 Hz) show the greatest improvement in glycemic control and metabolic health in individuals with type 2 diabetes compared to higher frequencies (>20 Hz), future studies are necessary to determine the most effective frequency and amplitude combination for WBV as a therapeutic in individuals with type 2 diabetes.

### 5.4. Use of Whole Body Vibration in Metabolic Syndrome

Metabolic syndrome is a combination of metabolic dysregulations that include increased adiposity, insulin resistance, dyslipidemia, and hypertension [60]. A few studies have explored the application of WBV in the context of metabolic syndrome. Notably, these investigations have uncovered compelling evidence of immediate and substantial alleviation of chronic pain and a marked enhancement in various aspects of the patient’s quality of life following rotational WBV (2 sessions per week for 5 weeks with increasing frequency and amplitude weekly; 5–14 Hz, 2.5–7.5 mm) [61,62]. In addition, among patients with metabolic syndrome, rotational WBV (10 sessions with increasing frequency and amplitude weekly; 5–14 Hz, 2.5–7.5 mm) enhanced balance and improved gait speed, both of which are parameters for overall functional capacity [63]. Interestingly, the specific frequency of WBV may be crucial when used in individuals with metabolic syndrome. Notably, employing rotational WBV (6 weeks; two sessions per week; 2.5–7.5 mm) with a range of low frequencies (5–16 Hz) compared to a single, fixed frequency (5 Hz) led to reductions in waist circumference and fat mass, along with an increase in bone content [64]. Additionally, a similar study demonstrated that using a range of lower frequencies (5–16 Hz) improved flexibility and decreased the rate of perceived exertion in individuals with metabolic syndrome [65]. Conversely, it is worth noting that no changes in blood pressure or heart rate were observed following 5 weeks of rotational WBV (10 sessions with increasing frequency and amplitude weekly; 5–14 Hz, 2.5–7.5 mm) [66]. In summary, the growing body of research investigating the application of WBV in the context of metabolic syndrome offers promising insights. Indeed, the current studies on metabolic syndrome have not utilized a standardized frequency or amplitude; instead, both parameters have been gradually increased throughout the investigation. There may be some benefit to this type of protocol, similar to a traditional exercise training regimen. However, the reported benefits cannot be attributed to a specific frequency or amplitude of vibration but rather provide insight into the beneficial effects of WBV. In fact, the WBV studies on metabolic syndrome highlighted in this review have utilized lower frequencies (5–14 Hz), which appear to exert a more favorable impact on both physical and physiological parameters within this patient population. Nevertheless, it is evident that further research within this specific patient population is warranted to provide a more comprehensive understanding and a deeper exploration of the potential benefits of WBV in managing metabolic syndrome and whether changing the frequency or amplitude would offer additional benefits.

While limited research has been conducted on the impact of WBV in a variety of metabolic diseases (presented above), it is worth noting that several metabolic conditions remain underexplored. These include, but are not limited to, type 1 diabetes, hyperlipidemia, homocystinuria, and cystic fibrosis-related diabetes. The notable benefits highlighted in this review underscore the importance of future investigations to explore the therapeutic application of WBV within the context of these additional metabolic disorders.

## 6. Conclusions and Future Directions

This comprehensive review delves into the intricate details of WBV, including the diverse array of WBV platforms and WBV regimens and the importance of dose by considering the interplay between vibration frequency and amplitude. In addition, this review sheds light on the favorable role of WBV across various metabolic diseases, including osteoporosis, overweight/obesity, type 2 diabetes, and metabolic syndrome. In an era where exercise modalities are rapidly evolving, WBV has emerged as a modern exercise mimetic, characterized by its increased accessibility and user-friendly nature, making it an increasingly prominent choice in contemporary fitness regimens. Table 1 provides a summary of the evidence regarding the optimal use of various types of WBV discussed in this review. When using vertical WBV, low frequency and low amplitude appear to be ideal for rehabilitation purposes, while high frequency with low amplitude in vertical WBV is best for neuromuscular activation. In addition, high frequency and amplitude may be more beneficial for weight loss, whereas low frequency with high amplitude can improve the quality of life in type 2 diabetes. When using rotational WBV, low frequency and low amplitude offer the lowest risk of side effects and show potential benefits for individuals with neck pain or vertigo. In addition, low frequency with high amplitude appears to be beneficial for improving metabolic health. Lastly, scant data were obtained using sonic wave WBV. Although frequency and amplitude were not presented, improvements in stability and mood in older individuals have been documented following sonic wave WBV. Nonetheless, it is important to emphasize that WBV holds therapeutic potential for individuals with metabolic conditions and can improve clinical outcomes; however, more research is certainly needed to identify the most effective WBV protocols to be used for each specific patient population.

## Figures and Tables

**Table 1:** Evidence Based WBV Protocol Review.

Type of Vibration	Frequency	Amplitude	Clinical Implications
*Vertical*	↓	↓	rehabilitation, bone health
	↑	↑	weight loss
	↓	↑	increased quality of life in type 2 diabetes
	↑	↓	neuromuscular activation
*Rotational*	↓	↓	lowest side effect profile, indicated for neck pain or vertigo
	↓	↑	reduction in HbA_1c_, reduction in waist circumference, and fat mass
*Sonic Wave*	?	?	stability and mood enhancements in elderly

Arrows indicate relative low (↓) or high (↑) frequency/amplitude.

## Data Availability

All data referred to in this review are available from the original publication.
